# Dyslipidemia Management in Patients with Coronary Artery Disease. Data from the POLASPIRE Survey

**DOI:** 10.3390/jcm10163711

**Published:** 2021-08-20

**Authors:** Piotr Jankowski, Paweł Kozieł, Małgorzata Setny, Marlena Paniczko, Maciej Haberka, Maciej Banach, Dirk De Bacquer, Guy De Backer, Kornelia Kotseva, David Wood, Zbigniew Gąsior, Karol Kamiński, Dariusz A. Kosior, Andrzej Pająk

**Affiliations:** 1 Department of Epidemiology and Health Promotion, School of Public Health, Medical Centre of Postgraduate Education, 01-813 Warsaw, Poland; 2 Institute of Cardiology, Jagiellonian University Medical College, 31-008 Kraków, Poland; tragez88@wp.pl; 3 Department of Cardiology and Hypertension with the Electrophysiological Lab, Central Research Hospital the Ministry of The Interior and Administration, 00-124 Warsaw, Poland; malgorzata.setny@cskmswia.gov.pl (M.S.); dkosior13@gmail.com (D.A.K.); 4 Department of Population Medicine and Lifestyle Diseases Prevention, Medical University of Bialystok, 15-089 Bialystok, Poland; Marlena.paniczko@umb.edu.pl (M.P.); karol.kaminski@umb.edu.pl (K.K.); 5 Department of Cardiology, Medical University of Silesia, 40-055 Katowice, Poland; mhaberka@op.pl (M.H.); zgasior@sum.edu.pl (Z.G.); 6 Department of Hypertension, Chair of Nephrology and Hypertension, Medical University of Lodz, 93-338 Lodz, Poland; maciejbanach@aol.co.uk; 7 Department of Public Health and Primary Care, Ghent University, 9000 Ghent, Belgium; dirk.debacquer@ugent.be (D.D.B.); guy.debacker@ugent.be (G.D.B.); 8 Imperial College Healthcare NHS Trust, London W2 1NY, UK; kornelia.kotseva@nuigalway.ie; 9 National Institute of Preventive Cardiology, National University of Ireland-Galway, H91 TK33 Galway, Ireland; d.wood2@imperial.ac.uk; 10 Cardiovascular Medicine, National Heart and Lung Institute, Imperial College, London W2 1NY, UK; 11 Faculty of Medicine, Medical College, Cardinal Stefan Wyszyński University, 01-938 Warsaw, Poland; 12 Department of Clinical Epidemiology and Population Studies, Institute of Public Health, Jagiellonian University Medical College, 31-066 Kraków, Poland; andrzej.pajak@uj.edu.pl

**Keywords:** cholesterol, coronary artery disease, hypercholesterolemia, secondary prevention

## Abstract

Lipid-lowering in patients with coronary artery disease (CAD) is related to a lower risk of cardiovascular events. We evaluated factors related to the management of hypercholesterolemia in patients with established CAD. Patients were interviewed 6–18 months after hospitalization for an acute coronary syndrome (ACS) or a myocardial revascularization procedure. Statins were prescribed at discharge to 94.4% of patients, while 68.1% of the patients hospitalized for an ACS were prescribed a high-dose statin. Hospitalization in a teaching hospital, percutaneous coronary intervention, cholesterol measurement during hospitalization and the male sex were related to prescription of statins at discharge. The intensity of lipid-lowering therapy in the post-discharge period increased in 17.3%, decreased in 11.7%, and did not change in 71.0% of the patients. The prescription of a lipid-lowering drug (LLD) at discharge (odds ratio 5.88 [95% confidence intervals 3.05–11.34]) and a consultation with a cardiologist (2.48 [1.51–4.08]) were related to the use of LLDs, while age (1.32 [1.10–1.59] per 10 years), loneliness (0.42 [0.19–0.94]), professional activity (1.56 [1.13–2.16]), and diabetes (1.66 [1.27–2.16]) were related to achieving an LDL cholesterol goal 6–18 months after discharge. In conclusion, health-system-related factors are associated with the LLD utilization, whereas mainly patient-related factors are related to the control of hypercholesterolemia following hospitalization for CAD.

## 1. Introduction

Patients with established coronary artery disease (CAD) are at a high risk of recurrent cardiovascular events [1]. Despite advances in pharmacological and invasive treatment, risk factors remain independent predictors of cardiovascular mortality in CAD patients [2]. One of the most important of these is hypercholesterolemia, while the use of lipid-lowering drugs is related to an improved prognosis [3]. The European Society of Cardiology guidelines consider lowering LDL cholesterol as the cornerstone of cardiovascular prevention [4,5]. Despite overwhelming evidence for the benefits of lowering cholesterol levels, especially when using statins, a majority CAD patients still have LDL cholesterol levels above the recommended goal [6,7,8,9]. Moreover, although survivors of an acute coronary syndrome should be prescribed high-dose statins, most patients take lower doses [10].

Recent surveys indicated that there is considerable potential for further improvements in the secondary prevention of CAD in European countries [11,12,13]. Many intervention methods aimed at improving secondary prevention in CAD patients have been proposed previously [14,15,16,17,18,19]. Additionally, several factors influencing the quality of secondary prevention in every-day practice have been identified [20,21,22]. Nevertheless, identifying the remaining barriers to effective risk factor control is essential to ensure the maximum benefits of prevention interventions. The aim of the present analysis was to investigate the factors affecting the management of hypercholesterolemia in patients with established CAD.

## 2. Materials and Methods

The POLASPIRE study was a cross-sectional, multicenter survey designed to evaluate the implementation of the European guidelines for secondary prevention of CAD by assessing both the control of the main risk factors and the prescription rates of cardioprotective medication in patients with established CAD. The survey has been described in detail elsewhere [8]. In brief, fourteen departments of cardiology from twelve different hospitals participated. Seven of the departments were situated in teaching hospitals and seven in municipal hospitals. The inclusion criteria included hospitalization for an acute coronary syndrome (ACS) or a myocardial revascularization procedure and age from ≥18 years to ≤80 years. Data collection using standardized methods and the same instruments in all centers were utilized by centrally trained research staff. Overall, 1236 patients were invited to participate in the study and their medical records were reviewed.

The follow-up interviews were completed 6–18 months after discharge from the hospital. A patient’s personal medical history, lifestyle and medications used were assessed using a standard questionnaire. The participants’ education was assessed based on the number of years of formal education completed. Self-perceived income was based on the answers to the question: “In your opinion, your family income is: very low, low, middle, high”. We looked at a measure of loneliness by including the question: “Do you have somebody with whom you share your problems or happiness?”. We constructed a socio-economic status (SES) summary score based on the different socio-economic components [23]. This score was the sum of the following sub-scores: educational level (primary school completed or less = 0, intermediate = 2, college/university = 4); perceived income (very low = 0, low = 2, intermediate = 4, high = 6); loneliness (yes = 0, no = 2); employment (yes = 2, no = 0); and being married (yes = 1, no = 0). Based on this summary score, varying from 0 to 15, we subdivided our sample in two groups: patients at a “low SES level” that had a summary score of ≤7, and patients at a “high SES level” that had a score of ≥8. The psychosocial characteristics of the patients were assessed on the basis of the Hospital Anxiety and Depression Scale [24].

Standard scales with a vertical ruler (SECA Medical Measuring Systems and Scales, Birmingham, UK) were used to complete measurements.

The scales were calibrated at the start of the survey. Height and weight were measured in a standing position without shoes and heavy outerwear. Body mass index (BMI) was calculated according to the following formula: BMI = weight [kg]/(height [m])^2^. Obesity was defined as a BMI ≥ 30 m/kg^2^. Smoking at the time of interview was defined as self-reported smoking verified by the concentration of breath carbon monoxide using a Smokerlyzer device (Model Micro+, Bedfont Scientific, Kent, UK). A high breath carbon monoxide was defined as 10 ppm or more. Blood pressure was measured twice, on the right arm in a sitting position after at least five minutes of rest with an automatic digital Omron Comfort M6 sphygmomanometers (OMRON Corporation, Kyoto, Japan). High blood pressure was defined as a blood pressure ≥140/90 mmHg or ≥140/85 mmHg in diabetic patients [4]. A fasting venous blood sample was acquired to measure plasma lipid and creatinine levels. The results of analyses performed no later than 12 h after blood collection were used for the purposes of the present report. A high cholesterol level was defined as an LDL cholesterol ≥1.8 mmol/L, whereas a high non-HDL cholesterol was defined as ≥2.6 mmol/L [4]. A high dose of statins was defined as atorvastatin in a dose of at least 40 mg per day or rosuvastatin in a dose of at least 20 mg per day. High-intensity cholesterol-lowering therapies were defined as a high-dose statin or any statin combined with the use of ezetimibe or a fibrate. All other lipid-lowering drug therapies were considered of “low or moderate intensity”. The glomerular filtration rate was defined using the MDRD formula [25].

The survey’s protocol was approved by the institutional Bioethics Committees. The study protocol complies with the ethical guidelines of the Declaration of Helsinki. All patients signed the informed consent form.

### Statistical Analysis

Qualitative variables were reported as percentages and continuous variables as means (standard deviation, SD) or median (interquartile range, IQR). The Pearson χ2 or Fisher’s exact test were used in the case of qualitative variables. The Shapiro–Wilk test was utilized to assess the normality of data. Normally distributed continuous variables were compared using the Student’s t-test. Variables without normal distributions were assessed using the Mann–Whitney U-test. Multivariable, stepwise logistic regression analysis was performed to assess the factors independently related to the dependent variables. The initial models comprised all variables mentioned in Table 1. A two-tailed *p*-value of less than 0.05 was regarded as statistically significant. Statistical analyses were completed using the STATISTICA 13 software (TIBCO Software, Palo Alto, CA, USA).

## 3. Results

### 3.1. In-Hospital Management

The medical records of 1236 patients were reviewed and included in the analyses, of whom 354 (29%) were females and 882 (71%) were males. The characteristics of the study population are presented in Table 1.

The patients’ total cholesterol levels were available in 997 (80.7%) hospital records, whereas LDL cholesterol measurements were found in 994 (80.4%) records. The only factors significantly related to the availability of total cholesterol values in the medical records were age and the index event (Table 2). Among patients hospitalized for ACS, the total cholesterol measurement was found in 633 (85.7%) hospital records, whereas LDL cholesterol measurements were available in 630 (85.3%) hospital records. The total cholesterol availability in the hospital records of patients hospitalized for ACS was independently related to age, hospitalization in a teaching hospital and hospitalization for a myocardial infarction (Table 2).

**Figure 1:**
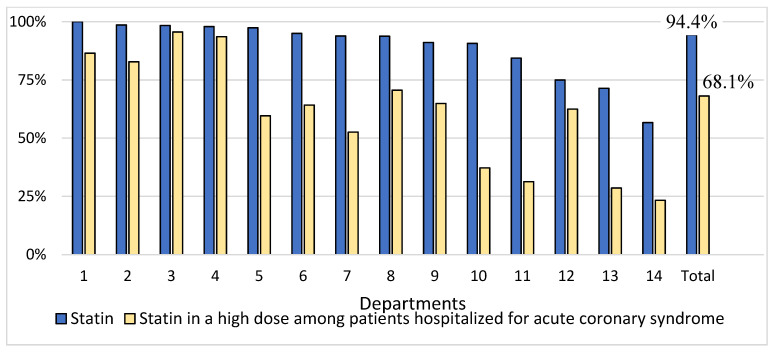
Proportions of patients prescribed a statin at discharge among all patients and statin in a high dose among those hospitalized for an acute coronary syndrome by departments.

A statin was prescribed at discharge to 1167 (94.4%) patients with significant variations between departments (*p* < 0.001; Figure 1). A total of 839 (67.9%) patients were prescribed a high-dose statin. The multivariable logistic analysis showed that hospitalization in a teaching hospital, percutaneous coronary intervention as an index event, cholesterol measurement during hospitalization and sex were independently related to the prescription of statins (Table 2). Among the patients prescribed a statin, 897 (77.1%) were prescribed atorvastatin, 221 (19.0%) were prescribed rosuvastatin, and 45 (3.9%) were prescribed simvastatin. The mean dose in the case of atorvastatin was 46.2 ± 27.2 mg per day, in the case of rosuvastatin it was 19.5 ± 11.1 mg per day, and in the case of simvastatin it was 24.7 ± 9.7 mg per day. Hospitalization for myocardial infarction was the only factor independently related to the prescription of atorvastatin compared to the other statins (odds ratio 2.88 [95% confidence intervals 2.09–3.99]). Among all studied patients, 41 (3.3%) patients were prescribed a fibrate and 16 (1.3%) were prescribed ezetimibe. No patient was prescribed any other lipid-lowering drug. Overall, 1171 (94.7%) of all patients were prescribed at least one lipid-lowering drug.

Among patients hospitalized for ACS, a statin at any dose was prescribed to 683 (92.4%) patients, whereas a high-dose statin was prescribed to 503 (68.1%) patients with a significant variation between departments (*p* < 0.001; Figure 1). Hospitalization for myocardial infarction, hospitalization in a teaching hospital, cholesterol measurement during hospitalization and sex were independently associated with the prescription of statins (Table 2). Among patients prescribed a statin, 549 (80.6%) were prescribed atorvastatin, 109 (16.0%) were prescribed rosuvastatin, and 23 (3.4%) were prescribed simvastatin. The mean doses of the drugs were atorvastatin at 48.2 ± 27.0 mg per day, rosuvastatin at 19.5 ± 11.3 mg per day, and simvastatin at 23.0 ± 8.2 mg per day. Among ACS patients, 19 (2.6%) were prescribed a fibrate and 10 (1.4%) were prescribed ezetimibe. Overall, 686 (92.8%) of the ACS patients were prescribed at least one lipid-lowering drug.

### 3.2. Management Following Discharge

Overall, 1034 patients (83.7%) attended the follow-up visit (12.0 ± 3.7 months after their discharge from the hospital). The patients who attended did not differ significantly from those who did not participate in the follow-up examination with respect to age (64.6 ± 8.5 years vs. 65.7 ± 8.3 years; *p* = 0.08), sex (males: 71.6% vs. 70.0%; *p* = 0.63), index event (*p* = 0.12), and proportion of patients hospitalized in a teaching hospital (0.36). In nine cases, the blood cholesterol level was not measured. Therefore, we analyzed data of 1025 study participants in all subsequent analyses. A total of 918 (89.6%) patients took a statin, and 803 (78.3%) took a high-dose statin. Among patients prescribed a statin, 659 (71.8%) were prescribed atorvastatin, 224 (24.4%) rosuvastatin, and 35 (3.8%) were prescribed simvastatin. The mean doses of statins were atorvastatin at 39.3 ± 19.6 mg per day, rosuvastatin at 20.4 ± 11.1 mg per day, and simvastatin at 23.1 ± 9.9 mg per day. Among those who did not change the statin they took between discharge and the follow-up examination, the mean dose of atorvastatin (*n* = 607) decreased by 7.0 (4.9–9.2) mg per day, whereas the mean doses of rosuvastatin and simvastatin did not change significantly. Among all the analyzed patients, 39 (3.8%) patients were prescribed a fibrate and 27 (2.6%) were prescribed ezetimibe. A statin plus a fibrate was used by 35 (3.4%) patients, while a statin plus ezetimibe was taken by 23 (2.2%) patients. Overall, 925 (90.2%) patients took at least one lipid-lowering drug. Among patients prescribed a lipid-lowering drug at the time of hospital discharge, 896 (91.4%) were taking a lipid-lowering drug at the time of the follow-up examination, whereas among those not prescribed any lipid-lowering drugs at the time of hospital discharge, 29 (64.4%) used at least one cholesterol-lowering agent (*p* < 0.001). The intensity of lipid-lowering therapy from discharge to the follow-up interview increased in 177 (17.3%) patients, decreased in 120 (11.7%) patients, and remained unchanged in 728 (71.0%) patients (Table 3). Table 4 presents factors independently related to the use of lipid-lowering drugs at the time of the follow-up examination.

The mean level of total cholesterol was 4.12 ± 1.12 mmol/L, LDL cholesterol 2.19 ± 0.95 mmol/L, HDL cholesterol 1.32 ± 0.38 mmol/L, non-HDL cholesterol 2.80 ± 1.08 mmol/L, and triglycerides 1.50 ± 0.99 mmol/L (median 1.29 mmol/L, interquartile range 0.95–1.77 mmol/L). Mean LDL cholesterol was 2.07 ± 0.86 mmol/L in patients taking atorvastatin, 2.10 ± 0.88 mmol/L in patients using rosuvastatin, 2.32 ± 0.92 mmol/L in patients using simvastatin, and 3.07 ± 1.13 mmol/L in those not using any statin. LDL cholesterol <1.4 mmol/L was found in 162 (15.8%) patients, <1.8 mmol/L in 382 (37.3%) patients, <2.6 mmol/L in 761 (74.2%), and <4.0 mmol/L in 967 (94.3%) patients, whereas non-HDL cholesterol <2.2 mmol/L, <2.6 mmol/L, <3.4 mmol/L, and <4.8 mmol/L was observed in 303 (29.3%), 519 (50.2%), 806 (77.9%), and 962 (93.0%) participants, respectively. The factors independently related to achievement of the recommended goals are presented in Table 4.

## 4. Discussion

The key finding of this study is that despite overwhelming evidence that lipid-lowering therapy decreases the risk of cardiovascular events in patients with coronary artery disease, 62.7% of study participants had LDL cholesterol levels above the recommended goal according to the 2016 guidelines [4]. Most of these patients probably failed to receive a proper evaluation and treatment, both in terms of pharmacotherapy as well as lifestyle modification, for dyslipidemia following hospitalization due to CAD. Overall, our results showed a sizeable potential for a further reduction in cardiovascular risk in patients with CAD through an improvement in hypercholesterolemia management. There is strong scientific evidence that the long-term survival of patients with CAD may be improved by providing optimal secondary prevention, including lipid-lowering treatment [4]. An even lower proportion of patients achieved the LDL cholesterol goal according to the most recent European Society of Cardiology guidelines [5].

Our results do not confirm the previous reports suggesting that the majority of patients discontinue their statin therapy within one year after initiation [26]. The intensity of lipid-lowering therapy declined in the small portion of patients during the post-discharge period. However, it was increased only in 17% of study participants, although most patients had high LDL cholesterol levels. Combination therapy was rarely applied. In particular, ezetimibe and proprotein convertase subtilisin/kexin type 9 inhibitors were underutilized [4,5,27,28]. Indeed, one critically important issue of the lack of efficacy of the lipid-lowering therapy is the very low application of combination therapy. Considering the new recommendations, immediate application of the combination therapy with statin and ezetimibe, and even triple therapy in selected cases, especially for the very-high-risk patients, might significantly improve effectiveness of the lipid-lowering treatment [5,29,30].

Hospitalization in a teaching hospital almost doubled the likelihood of a patient taking a lipid-lowering drug following discharge according to the report that analyzed data of patients hospitalized for CAD between 1996 and 1999 [31]. Our results suggest this association has not changed since the end of 20th century, despite multiple factors including several educational initiatives organized for physicians and profound changes in European societies induced by the collapse of the communist system in 1989 and the enlargement of the European Union. The Polish healthcare system has been in change for the last 20 years, both in terms of institutional changes and regulations regarding drug registration, prescription, and reimbursement. Importantly, the relative position of primary care physicians and specialists has evolved. In this respect, it is worth noting that the relationship between lipid-lowering drug use and practice settings has not changed significantly [31]. We showed a considerable variation in the use of lipid-lowering therapies among cardiac departments in addition to the previously found differences among countries [6]. The evidence suggests the variation has not decreased significantly over the last 20 years [32,33].

Our results suggest that specialized cardiology care is independently related to higher lipid-lowering drug utilization. There are several possible explanations for this finding. Firstly, patients consulted and not consulted by a cardiologist may differ with respect to a number of unrecognized factors, including greater compliance with the physicians’ recommendations. Secondly, cardiologists may dedicate more time to controlling cardiovascular risk factors compared to family doctors. Finally, a specialist usually has a greater authority than a family doctor and this could also partially explain the finding. The relationship between a consultation with a cardiologist and the proportion of patients with a cholesterol level at the recommended goal could help explain the lower risk of death among post-infarction patients who consulted a cardiologist [34].

Among patients analyzed in the Euroaspire V survey, no correlation was observed between a patient’s level of education and the LDL cholesterol level following hospitalization due to CAD [20]. Our results are concordant with the analysis conducted by De Bacquer et al., as there was no observed correlation between an individual’s level of education and ensuring the appropriate control of cholesterol level in our study group. We showed that a high socio-economic status and loneliness are independently related to the effectiveness of hypercholesterolemia management in patients with coronary artery disease. These results expand on earlier findings of the relationship between loneliness and risk factor control [20].

A lack of employment was independently associated with a high LDL cholesterol level. This might be due to financial barriers, but other factors (e.g., number of comorbidities) may also be responsible for this finding. In addition, control of hypercholesterolemia was related to smoking, obesity, and high blood pressure. This may underline the importance of inappropriate lifestyle modification and low adherence to prescribed therapies. Importantly, participation in a cardiac rehabilitation program was not independently related to the probability of LDL cholesterol levels at the recommended goal.

Finally, we found females to be prescribed a statin at discharge significantly less frequently. Females were also less likely to achieve the recommended non-HDL cholesterol level in the post-discharge period. The present results support the findings from the multicenter survey EUROASPIRE V [35]. This finding suggests the sex-gap in risk factor control of coronary patients is far from closing in many European countries. Further actions are needed to increase the awareness of the worse cholesterol control in female patients.

### Limitations

Besides the design of the study which precluded any consideration of causality, the present analysis has several other limitations. Firstly, we could not assess the association between hypercholesterolemia management and the risk of cardiovascular events. Secondly, our study participants were not representative of all coronary artery disease patients. The analyzed patients were limited to those who had experienced an acute CAD event or had undergone a myocardial revascularization procedure. Thirdly, we did not include patients aged over 80 years. Fourthly, we could not assess adherence to medications. The assessment of adherence would have increased the impact of our study. Conversely, an important advantage of our analysis is that our results are not based only on abstracted medical record data only, but involved face-to-face interviews and examinations using the same protocol and standardized methods and instruments. Thus, to the best of our knowledge, the current findings provide the most trustworthy data on cholesterol management for secondary prevention of CAD.

## 5. Conclusions

Health-system-related factors are associated with the utilization of lipid-lowering drugs, whereas mainly patient-related factors are related to the control of hypercholesterolemia following hospitalization due to coronary artery disease. Our results may help in the task of developing strategies to improve hypercholesterolemia management in patients with CAD.

## Figures and Tables

**Table 1:** Characteristics of the study group.

Variable	Median or Number
Age, years, median (IQR)	65.3 (59.9–71.3)
Sex	
Males, *n* (%)	882 (71.4)
Females, *n* (%)	354 (28.6)
Recruiting event	
Myocardial infarction, *n* (%)	481 (38.9)
Unstable angina, *n* (%)	258 (20.9)
PCI, *n* (%)	443 (35.8)
CABG, *n* (%)	54 (4.4)
Hospitalization in a teaching hospital, *n* (%)	903 (73.1)
Previous hospitalization for cardiovascular disease, *n* (%) ^a^	528 (51.5)
Duration of education, years, median (IQR) ^a^	12.0 (11.0–14.0)
Marital status ^a^	
Married, *n* (%)	750 (73.2)
Divorced/separated, *n* (%)	85 (8.3)
Widow/widower, *n* (%)	153 (14.9)
Never married, *n* (%)	37 (3.6)
Living alone, *n* (%) ^a^	161 (15.7)
Loneliness, *n* (%) ^a^	37 (3.1)
Household income ^a^	
High, *n* (%)	48 (4.7)
Medium, *n* (%)	655 (63.9)
Low, *n* (%)	274 (26.5)
Very low, *n* (%)	48 (4.6)
Employed, *n* (%) ^a^	316 (30.8)
High socio-economic status, *n* (%) ^a^	387 (37.8)
Participation in a cardiac rehabilitation program following discharge, *n* (%) ^a^	297 (29.0)
Regular physical activity	140 (13.7)
Physician specialty ^a^	
Cardiologist, *n* (%)	879 (85.8)
General practitioner, *n* (%)	880 (85.9)
Diabetologist, *n* (%)	111 (10.8)
Other physician, *n* (%)	28 (2.7)
No physician, *n* (%)	8 (0.8)
Smoking, *n* (%) ^a^	176 (17.2)
Obesity, *n* (%) ^a^	433 (42.2)
Diabetes, *n* (%) ^a^	408 (39.8)
High blood pressure, *n* (%) ^a,c^	433 (42.2)
High LDL cholesterol, *n* (%) ^a,d^	643 (62.7)
GFR < 60 mL/kg/1.73 m^2^, *n* (%) ^a^	185 (18.0)
Depression score, median (IQR) ^a,e^	5.0 (3.0–8.0)
Anxiety score, median (IQR) ^a,e^	6.0 (3.0–8.0)

Abbreviations: CABG, coronary artery bypass grafting; GFR, glomerular filtration rate; IQR, interquartile range; LDL, low-density lipoprotein; PCI, percutaneous coronary intervention. ^a^ Among subjects who participated in the follow-up examination. ^b^ Hospitalization before the recruiting event due to: coronary artery bypass grafting; percutaneous coronary intervention; acute coronary syndrome; chronic coronary syndrome; heart failure; stroke; or peripheral artery disease. ^c^ Blood pressure ≥140/90 mmHg or ≥140/85 mmHg in diabetics. ^d^ LDL cholesterol ≥1.8 mmol/L. ^e^ Based on the Hospital Anxiety and Depression Scale.

**Table 2:** Factors independently related to the availability of a total cholesterol measurement during hospitalization and to the prescription of a statin at discharge from the hospital.

Variable	Odds Ratio (95% Confidence Intervals)
**Factors Independently Related to the Availability of a Total Cholesterol Measurement During Hospitalization**	
*All patients (n = 1236)*	
Age, per 10 years	0.82 (0.68–0.99)
Index event	
Coronary artery bypass grafting	1.0
Percutaneous coronary intervention	1.89 (1.05–3.41)
Unstable angina	2.46 (1.31–4.61)
Myocardial infarction	4.96 (2.67–9.22)
*Patients hospitalized for an acute coronary syndrome (n = 739)*	
Age, per 10 years	0.72 (0.56–0.94)
Hospitalization in a teaching hospital	1.66 (1.09–2.54)
Hospitalization for myocardial infarction	1.91 (1.26–2.92)
**Factors independently related to the prescription a statin at discharge from the hospital**	
*Dependent variable: statin in any dose; all patients (n = 1236)*	
Hospitalization in a teaching hospital	2.90 (1.75–4.81)
Percutaneous coronary intervention as an index event	2.52 (1.32–4.84)
Total cholesterol measurement during hospitalization	2.27 (1.31–3.92)
Males vs. females	2.00 (1.21–3.31)
*Dependent variable: high-dose statin among patients hospitalized for an acute coronary syndrome (n = 739)*	
Hospitalization for myocardial infarction	3.82 (2.71–5.38)
Hospitalization in a teaching hospital	2.41 (1.70–3.40)
Total cholesterol measurement during hospitalization	2.51 (1.60–3.92)
Males vs. females	1.49 (1.04–2.14)

**Table 3:** Change in the lipid-lowering therapies from discharge to the follow-up examination.

Prescribed at Discharge	Used at the Time of Interview	*n* (%)
No drugs (*n* = 45)	No drugs	16 (35.6)
	Low/moderate-intensity therapy	4 (8.9)
	High-intensity therapy	25 (55.6)
Low/moderate-intensity therapy (*n* = 249)	No drugs	29 (11.8)
	Low/moderate-intensity therapy	72 (28.9)
	High-intensity therapy	148 (59.4)
High-intensity therapy (*n* = 731)	No drugs	55 (7.5)
	Low/moderate-intensity therapy	36 (4.9)
	High-intensity therapy	640 (87.6)

**Table 4:** Factors independently related to the use of at least one lipid-lowering drug and to achieving the recommended goal for LDL and non-HDL cholesterol in the post-discharge period.

Variable	Odds Ratio (95% Confidence Intervals)
**Factors Independently Related to the Use of at Least One Lipid-Lowering Drug in the Post-Discharge Period**	
*Prescription of a lipid-lowering drug at discharge excluded from the statistical model*	
At least one consultation with a cardiologist in the post-discharge period	2.45 (1.50–4.00)
Hospitalization in a teaching hospital	1.97 (1.22–3.17)
Professional activity	1.65 (1.00–2.72)
*Prescription of a lipid-lowering drug at discharge included in the statistical model*	
Prescription of a lipid-lowering drug at discharge	5.88 (3.05–11.34)
At least one consultation with a cardiologist in the post-discharge period	2.48 (1.51–4.08)
**Factors Independently Related to Achieving the Recommended Goal for LDL and non-HDL Cholesterol in the Post-Discharge Period**	
*Dependent variable: LDL cholesterol <1.8 mmol/L*	
Loneliness	0.42 (0.19–0.94)
Age, per 10 years	1.32 (1.10–1.59)
Employment	1.56 (1.13–2.16)
Diabetes	1.66 (1.27–2.16)
Statin prescribed at discharge	1.88 (1.00–3.57)
*Dependent variable: non-HDL cholesterol <2.6 mmol/L*	
CABG as an index event	0.52 (0.27–0.98)
Smoking	0.65 (0.45–0.92)
Obesity	0.73 (0.56–0.94)
High blood pressure	0.75 (0.58–0.98)
Age, per 10 years	1.31 (1.10–1.54)
Males vs. females	1.37 (1.03–1.83)
High socio-economic status	1.41 (1.06–1.88)
Diabetes	1.42 (1.09–1.86)

## Data Availability

Data available on request only for scientific purposes.
